# HCoV‐HKU1 and TMPRSS2 interaction: uncovering mechanisms of viral invasion

**DOI:** 10.1002/mco2.70051

**Published:** 2025-01-10

**Authors:** Zehan Pang, Huahao Fan, Xiaolong Xu

**Affiliations:** ^1^ College of Life Science and Technology Beijing University of Chemical Technology Beijing China; ^2^ Beijing Hospital of Traditional Chinese Medicine Capital Medical University Beijing China

1

Three recent studies published in *Cell* have revealed the intricate interaction between HCoV‐HKU1 and transmembrane serine protease 2 (TMPRSS2). These studies not only pave the way for a deeper understanding of the mechanism of viral invasion but also offer new perspectives on antiviral therapies against coronavirus.^1^, ^2^, ^3^

The spike (S) glycoprotein of coronaviruses facilitates membrane fusion with host cells, enabling the viral genome delivery. Identified entry receptors for coronavirus include angiotensin‐converting enzyme 2 (ACE2) for SARS‐CoV, SARS‐CoV‐2, HCoV‐NL63, and bat‐derived MERS ‐related coronaviruses; aminopeptidase N for HCoV‐229E, CcoV‐HuPn‐2018, PDCoV, TGEV/PRCV, and PEDV; dipeptidyl peptidase 4 for MERS‐CoV and HKU4‐related MERS‐related coronaviruses; and TMEM106B, which mediates ACE2‐independent entry of SARS‐CoV‐2 (Figure 1A).^1^

**FIGURE 1:**
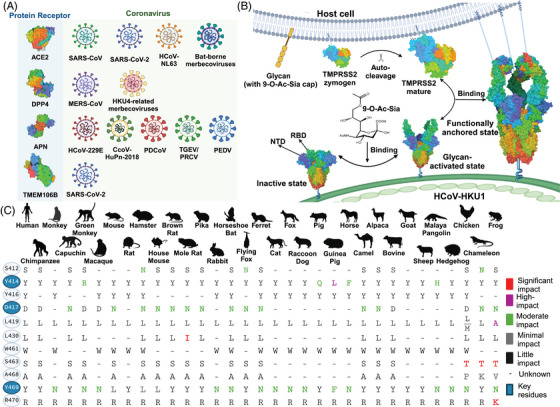
Synergistic mechanism of transmembrane serine protease 2 (TMPRSS2) and 9‐O‐acetylated sialosides (9‐O‐Ac‐Sia) during HCoV‐HKU1 enter cell, and the potential risk of cross‐species transmissibility of HCoV‐HKU1. 9‐O‐Ac‐Sia and TMPRSS2 act as the glycan receptor determinant and the major protein receptor for HCoV‐HKU1, respectively, and they facilitate the viral entry process. Some key positions on the interactive interface between HCoV‐HKU1 RBD and TMPRSS2 determine the possibility of the virus entering the cell, and some sites are conserved in mammals. (A) A summary figure of the currently known coronavirus protein receptors. They are ACE2 for SARS‐CoV, SARS‐CoV‐2, HCoV‐NL63 and Bat‐borne Merbecoviruses; APN for HCoV‐229E, CcoV‐HuPn‐2018, PDCoV, TGEV/PRCV and PEDV; DPP4 for MERS‐CoV and HKU4‐related Merbecoviruses; TMEM106B is a receptor mediating ACE2‐independent SARS‐CoV‐2 cell entry. ACE2: angiotensin‐converting enzyme 2; APN: aminopeptidase N; DPP4: dipeptidyl peptidase 4. (B) When 9‐O‐Ac‐Sia binds to the N‐terminal domain (NTD), the HCoV‐HKU1 S trimer transits from the inactive state to the glycan‐activated state that exposes the receptor‐binding domain (RBD). RBD is recognized and bound by matured‐TMPRSS2 which undergoes autocleavage. Subsequently, the RBD forms a functional anchor conformation, facilitating the invasion of target cells by the virus. (C) Comparison of key residues of TMPRSS2 in humans and 33 different species of animals (including Primates, Rodentia, Lagomorpha, Chiropteran, Carnivora, Perissodactyla, Artiodactyla, Pholidota, Erinaceomorpha, Galliformes, Lacertiformes, Anura), with Y414, D417, and Y469 being the key residues (marker with blue) most relevant to HCoV‐HKU1 host tropism. The sites were also coloured for mutations according to the strength of their ability to influence the binding of TMPRSS2 to the HCoV‐HKU1 RBD. Red: nearly incapacitated; Purple: high impact on binding capacity; Green: moderate impact on binding capacity; Gray: minimal effect on binding capacity; Black: little to no effect on binding capacity; “‐”: Unknown information.

HCoV‐HKU1, a seasonally transmitted β‐coronavirus,^1^ is known to cause common colds, respiratory infections, and serious complications.^1^, ^2^ In 2019, Hulswit et al. identified 9‐O‐acetylated sialic acids as receptors for HCoV‐HKU1.^4^ In 2023, Saunders et al. revealed that TMPRSS2 is the direct entry receptor for HCoV‐HKU1.^3^, ^5^ TMPRSS2 plays a crucial role in the proteolytic activation of the S glycoprotein of various coronaviruses, including SARS‐CoV‐2, SARS‐CoV, MERS‐CoV, and HCoV‐229E, as well as influenza virus hemagglutinins.^1^ After SARS‐CoV‐2 binds to ACE2 and attaches to the cell surface, TMPRSS2 or other proteases cleave the S2’ site on the spike protein, facilitating the necessary conformational change. Notably, TMPRSS2 is characterized by its protease activity and does not contribute to the endocytosis of SARS‐CoV‐2. Conversely, during HCoV‐HKU1 infection, TMPRSS2 facilitates spike‐mediated membrane fusion, even when its proteolytic activity is impaired. This distinction highlights a different role for TMPRSS2 in the entry mechanism of HCoV‐HKU1 compared to SARS‐CoV‐2.^5^ Three recent studies published in *Cell* detailed the comprehensive explanations of 9‐O‐acetylated sialosides (9‐O‐Ac‐Sia) and TMPRSS2 facilitating HCoV‐HKU1 entry and evaluated the species tropism of HCoV‐HKU1.^1^, ^2^, ^3^

McCallum et al.^1^ optimized a human TMPRSS2 (hTMPRSS2) expression vector that includes the T447C mutation, a cleavable N‐terminal SUMO fusion, and an N249‐linked glycosylation motif, significantly enhancing enzyme activity and yield in Expi293 cells. Additionally, they expressed the HKU1A receptor‐binding domain (RBD) and elucidated the structures of TMPRSS2 S441A and RBD using cryo‐electron microscopy (cryo‐EM). Their finding revealed that electrostatic contacts, salt bridges, van der Waals interactions, and hydrogen bonds mediated the interaction between these proteins. Wang et al.^2^ expressed and purified the ectodomain of HCoV‐HKU1A S and hTMPRSS2, identifying three states of HCoV‐HKU1 S: the inactive state, the glycan‐activated state, and the functionally anchored state (Figure 1B). In the inactive state, 42% of the S trimers exhibited up‐conformations.^2^ When 9‐O‐Ac‐Sia binds to the N‐terminal domain (NTD), the S trimer transitioned to the glycan‐activated state, increasing the up‐conformation from 42% to 73%. The transition involved a 20° inward rotation of the NTD and a 92° upward rotation of the RBD, facilitating binding to TMPRSS2 and enabling HCoV‐HKU1 to enter host cells. After binding with TMPRSS2 and 9‐O‐Ac‐Sia, the HCoV‐HKU1 S trimer formed four conformations of functional anchoring states, including 1up with 1TMPRSS2 (11.5%), 2up with 2TMPRSS2 (37.4%), 3up with 2TMPRSS2 (11.8%), and 3up with 3TMPRSS2 (39.3%).^2^ Fernández et al. found that TMPRSS2 maintains autolytic activation, allowing autocleavage to achieve a mature active conformation, and enhancing its binding affinity to the RBD.^1^, ^3^ Nanobody A07 can inhibit TMPRSS2's proteolytic activity, thereby reducing HCoV‐HKU1 infection.^3^, ^5^

Mutant binding experiments and key site mutation analyses by Wang et al. and McCallum et al. identified key interaction sites are Y414, W461, Y469, and R470 on TMPRSS2, as well as H488, E505, V/Y509, L510, W515, R517, and Y528 on RBD.^1^, ^2^ These interaction sites are highly conserved in the HCoV‐HKU1A and HCoV‐HKU1C strains, distinguishing them from other coronaviruses such as SARS‐CoV, SARS‐CoV‐2, and MERS‐CoV^2^.

The zoonotic potential of coronaviruses raises significant concerns. McCallum et al. analyzed the binding sites on TMPRSS2, while Wang et al. aligned TMPRSS2 sequences from 23 species, and Fernández et al. aligned 201 mammalian TMPRSS2 sequences. They found that these sequences are predominantly conserved among mammals (with significant differences in Y414, D417, and Y469 in some species, such as macaques, mice, hamsters, and ferrets), partially conserved in reptiles and birds, and less conserved in amphibians and other vertebrates.^1^, ^2^, ^3^ Mutations at key sites on hTMPRSS2 to other species, such as pig‐TMPRSS2 (Y414Q) and monkey‐TMPRSS2 (Y469N), resulted in a reduced or abolished binding activity with RBD.^1^, ^2^ Additionally, the HCoV‐HKU1 pseudovirus could enter TMPRSS2‐overexpressing cells from primates, rodents, artiodactyl, lagomorph, and chiropteran, but not from green monkeys, mole rats, ferrets, and horseshoe bats.^1^ Fernández et al. further confirmed the importance of these residues through membrane fusion assays and pseudovirus infection experiments^3^ (Figure 1C). The Y414 and Y469 residues of TMPRSS2 flank the L521 and P522 residues of the spike protein, forming a clamp‐like hydrophobic core. Mutations at these positions can disrupt the critical TMPRSS2‐spike interactions. Additionally, the D417 site on TMPRSS2 varies among species, typically comprising non‐charged residues, whereas hTMPRSS2 features polar or non‐polar residues. Furthermore, the W461 and R470 sites on hTMPRSS2 engage in hydrophobic interactions and a hydrogen bond network with the spike protein, respectively. Variations at these sites in other species can disrupt these essential interactions between TMPRSS2 and the spike protein.^2^, ^3^ These findings highlight the zoonotic and rodent origin hypotheses of HCoV‐HKU1.

The aforementioned studies elucidate the synergistic mechanisms of TMPRSS2 and 9‐O‐Ac‐Sia during HCoV‐HKU1 entry,^1^, ^2^, ^3^, ^5^ laying a theoretical groundwork for the development of pharmaceuticals, antibodies, and vaccines.^1^, ^2^ In response to selective pressures from antiviral therapies, viruses may evolve to harbour drug‐resistant mutations. Protease inhibitors, such as camostat, and antibodies targeting TMPRSS2 could potentially suppress protease activity, providing a broad‐spectrum antiviral effect, and help address the scarcity of effective treatments for HCoV‐HKU1 and simultaneously reduce the likelihood of drug‐resistant viral strains emerging. Nevertheless, inhibitors and antibodies might produce off‐target side effects, resulting in unforeseen consequences. Advanced computational methods can be employed to predict drug targets and cell‐based protein arrays can effectively detect antibody targets.

Furthermore, the studies offer valuable insight into the entry mechanism of coronavirus.^2^, ^3^ Revealing the interaction between HCoV‐HKU1 and TMPRSS2 is essential for preventing and treating the diseases associated with coronaviruses in the future. HCoV‐HKU1, which originates from rodents, has the potential to infect humans through domestic animals that may act as intermediate hosts, although the specific species involved remain unidentified. Analysis of TMPRSS2 homology reveals that HCoV‐HKU1 can recognize receptors across various species, thereby facilitating interspecies transmission. The virus is characterized by a high frequency of recombination and a significant mutation rate, which are key evolutionary factors that enable coronaviruses to adapt efficiently to a range of hosts and environments, thus promoting zoonotic transmission. Furthermore, ecological factors such as human exploration, travel, modern agricultural practices, and urbanization contribute to the spread of these viruses from their natural reservoirs to other animals and humans, increasing the risk of zoonotic infections.

Human coronaviruses must replicate within humans to acquire adaptive mutations that counteract host restriction factors. As the viruses spread across species or within humans, they undergo continuous mutation and recombination processes, which may enhance virulence and facilitate immune evasion. Understanding virus‐host interactions is crucial for preventing potential coronavirus spillovers and preparing for the future outbreaks.

## AUTHOR CONTRIBUTIONS

**Zehan Pang**: Writing–original draft preparation; review and editing; visualization. **Huahao Fan**: Conceptualization; writing–original draft preparation; review and editing; funding acquisition. **Xiaolong Xu**: Writing–review and editing; funding acquisition. All authors have read and approved the final manuscript.

## CONFLICT OF INTEREST STATEMENT

The authors declare no conflicts of interest.

## ETHICS STATEMENT

Not applicable.

## Data Availability

Not applicable.
